# Alcohol-induced brain deficit in alcohol dependence

**DOI:** 10.3389/fneur.2022.1036164

**Published:** 2022-10-25

**Authors:** Yanping Wang, Bo Sun

**Affiliations:** ^1^Department of Neurosurgery, The Affiliated Huai'an No. 1 People's Hospital of Nanjing Medical University, Huai'an, China; ^2^Department of Neurology, The Affiliated Huai'an No.1 People's Hospital of Nanjing Medical University, Huai'an, China

**Keywords:** alcohol dependence, percent amplitude of fluctuation, receiver operating characteristic, cerebellar-visual-orbitofrontal circuit, functional MRI

## Abstract

Although numerous adverse effects of alcohol addiction on health, behavior, and brain function were widely reported, the neurobiological mechanism of alcohol dependence remains largely unknown. In this study, a total of twenty-nine patients with alcohol dependence and twenty-nine status-matched normal controls (NCs) were recruited. Percent amplitude of fluctuation (PerAF) was applied to identify alcohol-related brain activity deficits. We found that alcohol dependence was associated with widespread differences in the left orbitofrontal cortex, right higher visual cortex, right supramarginal gyrus, right postcentral gyrus, and bilateral cerebellum posterior lobe with decreased PerAF, but no brain areas with increased PerAF differences were found. ROC curve showed that decreased PerAF revealed extremely high discriminatory power with a high AUC value of 0.953, as well as a high degree of sensitivity (96.6%) and specificity (86.2%), in distinguishing patients with alcohol dependence from NCs. In the alcohol dependence group, the amount of daily alcohol consumption showed significant negative correlations with the right cerebellum posterior lobe and right higher visual cortex. These findings suggest that the cerebellar-visual-orbitofrontal circuit was disturbed by alcohol dependence. The proposed new method of PerAF may be served as a potential biomarker to identify the regional brain activity deficits of alcohol dependence.

## Introduction

Alcohol use is a leading risk factor for disease burden worldwide, accounting for nearly 10% of global deaths among populations aged 15–49 years, and poses dire ramifications for future population health in the absence of policy action today (1). Alcohol addiction, a serious public problem, is characterized by morbid, excessive, and continued alcohol consumption. It may have substantial heritability (2) and may lead to high morbidity or mortality. Although alcohol consumption would relieve some negative emotions, numerous adverse effects of alcohol addiction on health, behavior, and brain function are widely reported, which may bring cancer, vehicle accidents, cognitive disorder, and liver cirrhosis (3, 4).

Rapid advances in neuroimaging techniques promote researchers to further explore the neurobiology mechanism of alcohol addiction and its consequence (5, 6). Brain imaging methods have been widely used to evaluate the relationships between the adverse effects of excessive alcohol use on regional brain areas, neural circuitry, and behavior in humans (5). Alcohol dependence has been found to be related to regional activity deficits in several brain areas (3, 5, 7, 8). Resting-state functional MRI (rs-fMRI) has developed rapidly in recent years, enabling it to identify these changes and their relationships with addictive symptoms (9). Therefore, the rs-fMRI may be an applicable method to address the alteration of regional brain activity associated with alcohol dependence (10). Although an increasing number of studies have applied to insight into the neuroimaging findings of alcohol dependence (3, 5, 7, 8, 11, 12), its neurobiological mechanism remains largely unknown.

Recently, the amplitude of low-frequency fluctuations (ALFFs) has been widely applied to studying the regional brain activity of alcohol dependence (3). It has served as an early biological biomarker to monitor the spontaneous neuronal fluctuation of regional brain activity of psychiatric disorders, such as alcohol dependence due to its high test-retest reliability (3, 10, 13–18). However, this method was easy to be influenced by cardiac noise and physiological high-frequency respiratory. Although the rs-fMRI design did not have an explicit task, a similar metric to the percentage of signal change for the rs-fMRI data can be formulated, by calculating the percentage of blood oxygen level-dependent (BOLD) fluctuations relative to the mean BOLD signal intensity for each time series, namely, Percentage Amplitude Fluctuation (PerAF). The PerAF could avoid the confounding mixture from voxel-specific fluctuation amplitude in fractional ALFF and seems to be a promising metric of voxel-level spontaneous BOLD activity. The PerAF has been proven to have the best reliability relative to regional homogeneity, ALFF, and degree centrality (19–21). Therefore, the PerAF method may be more sensitive to describe the alcohol dependence-induced regional brain activity alternation relative to other rs-fMRI methods. However, its application to alcohol dependence has not been studied.

Excessive and continued alcohol consumption could lead to a variety of neuroanatomical and neurochemical alternations in the neural circuitry, monoamine systems, neuropeptide systems, ion channels, and amino acid neurotransmitter systems (22). These changes are mainly reflected in altered regional brain activity or neural circuitry (23). Therefore, we hypothesized that alcohol dependence was associated with alcohol-induced alternation in some specific regional brain areas or neural circuitry. To test the hypothesis, we utilized the PerAF method to evaluate the alternation of regional brain activity in patients with alcohol dependence relative to normal controls (NCs), which may yield promoting us to insight into the neurobiological mechanism underlying alcohol dependence.

## Materials and methods

### Subjects

A total of twenty-nine patients with alcohol dependence (20 males, 9 females; age, 48.62 ± 6.81 years; education, 9.52 ± 2.87) and twenty-nine age-, sex-, and education-matched NCs (18 males, 11 females; age, 48.48 ± 7.05 years; education, 8.48 ± 3.1 years at school) were recruited from our hospital and community in this study. All patients should meet the diagnostic criteria of alcohol use disorders based on DSM-IV. The data of severity of the alcohol dependence questionnaire (SADQ), alcohol use disorders identification test (AUDIT), and life history (psychiatric disorders, years of drinking, and daily alcohol consumption) were recorded.

All recruited subjects have not taken any treatment by medications before. All subjects did not report any history of other substance dependence or abuse, pathological brain lesions or head trauma, and foreign implants, as well as any history of neurological disorders or psychiatric illnesses (5, 7, 8). This study was approved by the Ethical Committee of our hospital. All subjects finished their written informed consent.

### MRI data collection

We used a 3.0-Tesla MR scanner (Trio, Siemens, Erlangen, Germany) to finish the rs-fMRI session. First, a total of 176 slices of high-resolution anatomical volumes in a sagittal orientation [repetition time/echo time (TR/TE) = 1,950/2.3 ms, gap/thickness = 0/1 mm, field of view (FOV) = 244 mm × 252 mm, acquisition matrix = 248 × 256, and flip angle = 9°] were collected. Then, a total of 240 functional volumes (TR/TE = 3,000/25 ms, gap/thickness = 0.5/5.0 mm, flip angle = 90°, acquisition matrix = 32 × 32, and FOV = 210 mm × 210 mm) were collected. Before the rs-fMRI scan, all subjects were asked to go to the toilet and rest quietly for at least 30 min. During the rs-fMRI scan, all subjects should wear black blinders and sponge earplugs. All subjects were told to relax and think nothing.

### Data analysis

The rs-fMRI data preprocessing was analyzed using RESTplus version 1.2 (http://www.restfmri.net) toolbox, including the form transformation, removing the first 10 functional volumes, slice timing and head motion correction, spatial normalization, smooth with 6 × 6 × 6 mm full-width Gaussian kernel, linear detrending, and filter (low frequency, 0.01–0.08 Hz). In our study, no subjects have a head motion with more than 1.5 mm maximum translation in any direction and/or more than 1.5° of rotations. The Friston 24 head motion parameters (6 head translation, 6 head rotation, and 12 corresponding squared items) were used to regress out the head motion effects (24–27). The remaining functional volumes were spatially normalized to Montreal Neurological Institute (MNI) space. All volumes were resampled and transformed the resolution into 3 × 3 × 3 mm^3^. The covariates of head-motion parameters, white matter, global mean signal, and cerebrospinal fluid signal were removed using linear regression analysis. The PerAF method is the percentage of the resting-state frequency domain of the BOLD signal relative to the mean signal intensity of each time series. Finally, the PerAF, mPerAF, and z-transformation of zPerAF were generated.

### Statistical analysis

Unpaired two-sample *t*-tests were applied to compare the differences in demographic characteristics (age, years of education, and AUDIT score) between patients with alcohol dependence and NCs. The sex difference was calculated using the chi-square (χ^2^) test. The threshold of *p* < 0.05 was used to determine the differences. The statistical analysis was performed using IBM Statistical Package for the Social Sciences version 21.0 (SPSS 21.0). For rs-fMRI data, first, one sample *t*-tests were used to compare the within-group differences in brain areas for alcohol dependence or NCs, respectively. The threshold of voxel-wise *p* < 0.001 and cluster-level *p* < 0.001 was used to determine the between-group differences for one-sample *t-*tests, which was corrected by a false discovery rate (FDR). Then, a two-sample *t*-test was used to analyze the PerAF differences in regional brain activities between alcohol dependence and NCs, which was corrected by AlphaSim using the threshold of voxel-wise *p* < 0.01 and cluster-level *p* < 0.05 (minimum continuous cluster voxel volumes ≥1,080 mm^3^). Age, years of education, and sex were utilized as nuisance covariates of no interest for these analyses.

Recently, the receiver operating characteristic (ROC) curve was increasingly used to identify whether one imaging parameter could be served as a potential biomarker to discriminate between two different groups (10, 13, 15, 28–30). In this study, we used the ROC curve to evaluate the discriminatory ability of these regional brain deficits in distinguishing patients with alcohol dependence from NCs. We applied Pearson correlation to evaluate the correlations between behavioral data and regional brain deficits. The threshold of *p* < 0.05 was used to determine the differences.

## Results

### Sample characteristics

The demographic results of the two groups are presented in Supplementary Table 1. Patients with alcohol dependence differed from the NCs in sex (χ^2^ = 0.31, *p* = 0.581), age (*t* = 0.10, *p* = 0.94), and education level (*t* = 1.32, *p* = 0.19). NCs showed lower AUDIT scores than that alcohol dependence (*t* = 20.35, *p* < 0.001). The mean duration of drink history, mean SADQ score, and mean daily alcohol consumption was (27.93 ± 10.28) years, (20.34 ± 6.89), and (239.66 ± 107.22) ml in the patient group, respectively.

### PerAF differences

**Figure 1:**
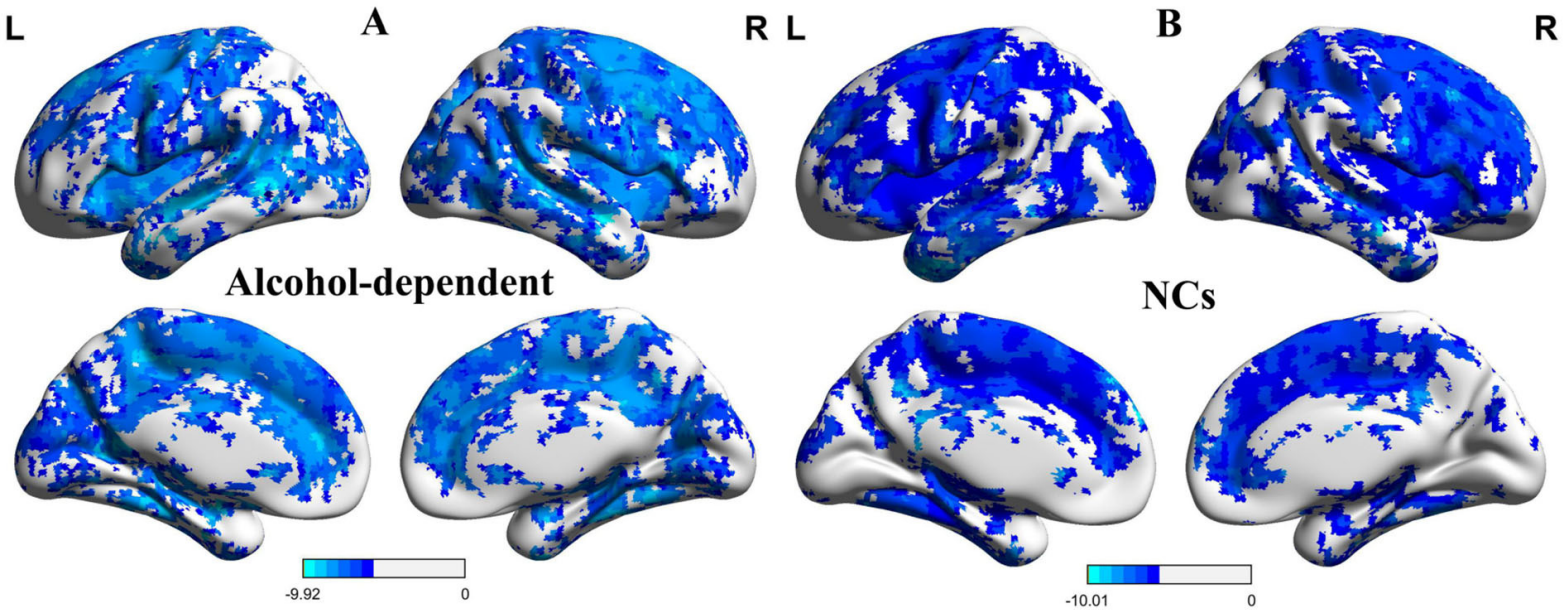
One sample *t*-test differences of alcohol dependence and NCs in PerAF maps. **(A)** One sample *t*-test differences of alcohol dependence. **(B)** One sample *t*-test differences of NCs.R, right; L, left; NCs, normal controls; PerAF, percent amplitude of fluctuation.

**Figure 2:**
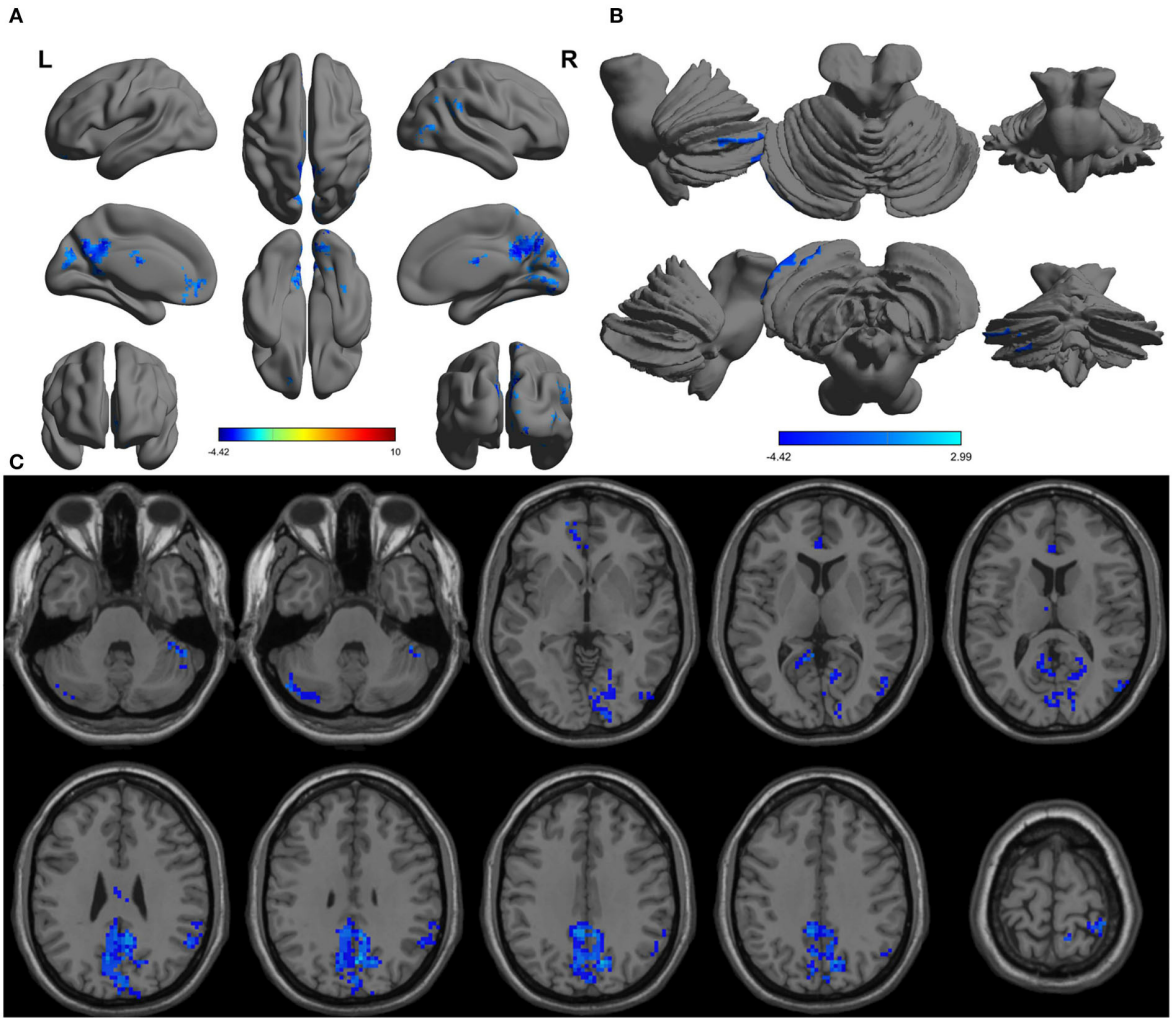
Altered PerAF in patients with alcohol dependence relative to NCs. **(A)** Cerebral viewer without cerebellum, **(B)** cerebral viewer in cerebellum, and **(C)** axial view with whole brain. Blue color, decreased PerAF areas. R, right; L, left; PerAF, percent amplitude of fluctuation; NCs, normal controls.

The one-sample statistical maps showed that the covered PerAF differences in regional brain areas of patients with alcohol dependence (Figure 1A) were smaller than that of NCs (Figure 1B). The between-group statistical maps exhibited that, compared with NCs, patients with alcohol dependence showed decreased PerAF differences in the right supramarginal gyrus (BA 40), left orbitofrontal cortex (Brodmann's area, BA 11), right higher visual cortex (BA 18, 19, and 37), right postcentral gyrus (BA 2), and bilateral cerebellum posterior lobe, but no increased PerAF differences in brain areas were found between patients with alcohol dependence and NCs (Table 1; Figures 2A–C).

**Table 1:** The PerAF differences between patients with alcohol dependence and NCs.

**Brain regions of peak coordinates**	**R/L**	**BA**	**Voxel volume (mm^3^)**	**t-score of peak voxels**	**MNI coordinates**
					***X, Y, Z***
Cerebellum Posterior Lobe	L	N/A	1,728	−3.01	−48 −75 −36
Cerebellum Posterior Lobe	R	N/A	1,269	−2.99	51 −48 −33
Medial Frontal Gyrus	L	11	2,646	−3.11	−12 45 −15
Lingual Gyrus, Cuneus	R	18	22,977	−4.42	12 −87 −9
Middle Occipital Gyrus, Middle Temporal Gyrus	R	18, 19, 37	1,593	−2.84	57 −72 15
Supramarginal Gyrus	R	40	2,511	−3.01	57 −51 24
Postcentral Gyrus	R	2	1,080	−2.97	42 −42 66

The statistical threshold was set at a corrected significance level of individual two-tailed voxel-wise *p* < 0.05 using an AlphaSim corrected threshold of cluster *p* < 0.05.

PerAF, percent amplitude of fluctuation; NCs, normal controls; R, right; L, left; BA, Brodmann's area; MNI, Montreal Neurological Institute; N/A, not applicable.

### ROC curve

**Figure 3:**
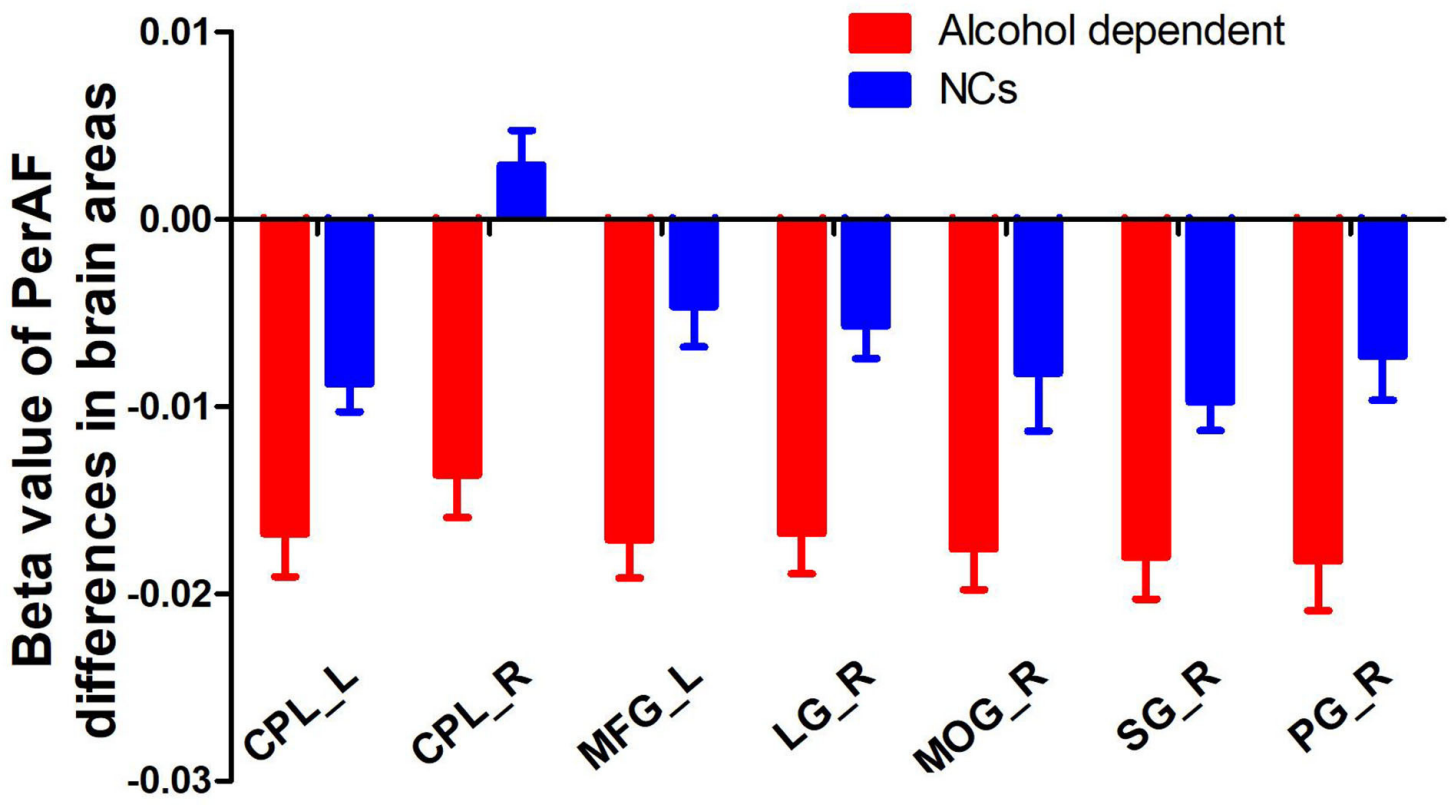
Beta value of between-group differences in PerAF in regional brain areas. PerAF, percent amplitude of fluctuation; NCs, normal controls; R, right; L, left; CPL, cerebellum posterior lobe; MFG, medial frontal gyrus; LG, lingual gyrus; MOG, middle occipital gyrus; SG, supramarginal gyrus; PG, postcentral gyrus.

**Figure 4:**
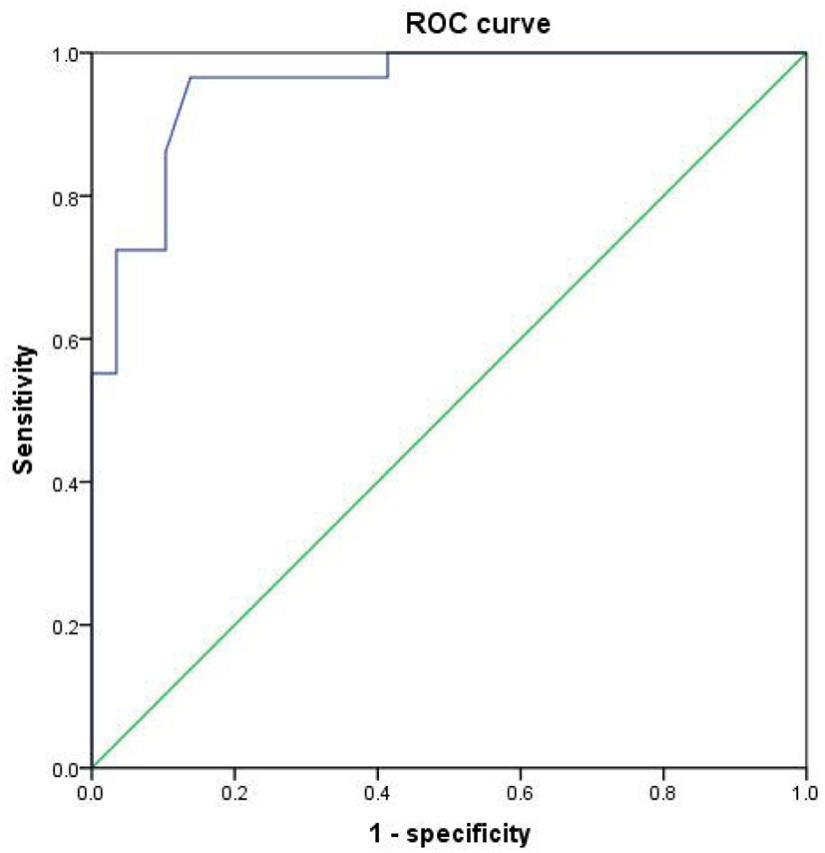
ROC curve of PerAF differences in brain areas. ROC, receiver operating characteristic; PerAF, percent amplitude of fluctuation.

Since the regional brain areas that exhibited between-group differences may be potential biomarkers to distinguish patients with alcohol dependence from NCs, we extracted the mean PerAF values of these regional brain areas for ROC curve analysis (Figure 3). Our findings suggest that these specific regional brain areas had a high degree of discriminatory power with an extremely high AUC value of 0.953 (Figure 4). Further diagnostic analysis exhibited a high degree of sensitivity (96.6%) and specificity (86.2%) of the PerAF with a cutoff value of 0.542.

### Pearson correlation analysis

**Figure 5:**
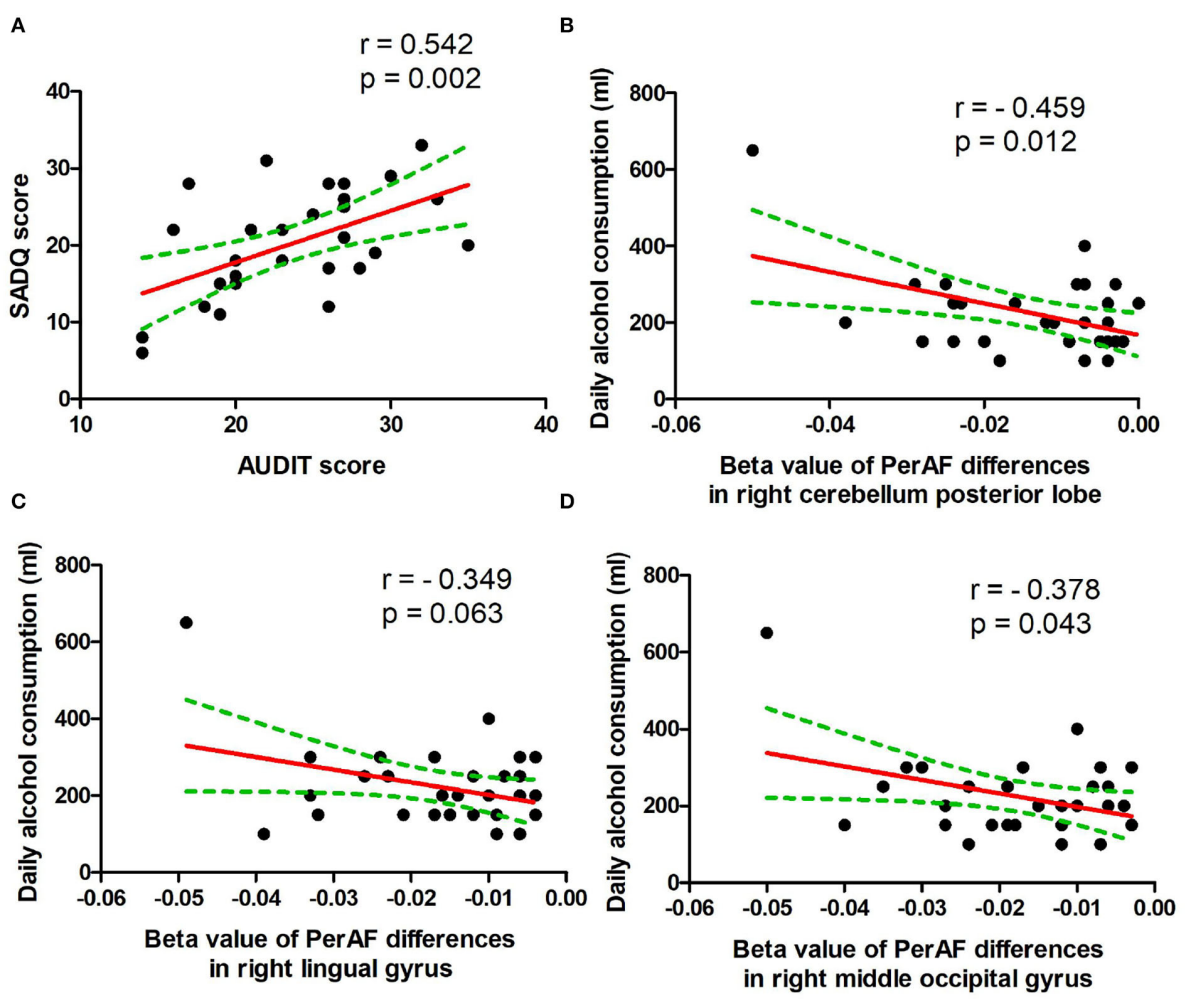
Pearson's correlation. **(A)** Correlation between SADQ and AUDIT, **(B)** correlation between daily alcohol consumption and right cerebellum posterior lobe, **(C)** correlation between daily alcohol consumption and right lingual gyrus, and **(D)** correlation between daily alcohol consumption and right middle occipital gyrus. SADQ, severity of alcohol dependence questionnaire; AUDIT, alcohol use disorders identification test; PerAF, percent amplitude of fluctuation.

In the patient group, several correlation analyses between the demographic results and the PerAF values of the regional brain areas that exhibited differences between patients with alcohol dependence and NCs were calculated. Our data showed several significant correlations in the patient group (Figures 5A–D). The SADQ score exhibited a positive correlation with AUDIT score (*r* = 0.542, *p* = 0.002; Figure 5A).

The amount of daily alcohol consumption showed significant negative correlations with PerAF values of right cerebellum posterior lobe (*r* = − 0.459, *p* = 0.012; Figure 5B) and right higher visual cortex (right lingual gyrus, *r* = − 0.349, *p* = 0.063, Figure 5C; right middle occipital gyrus, *r* = −0.378, *p* = 0.043, Figure 5D). Furthermore, the correlation between the right cerebellum posterior lobe and the amount of daily alcohol consumption was still significant even after Bonferroni correction.

## Discussion

This study is the first to utilize the proposed PerAF method to identify alcohol-induced regional brain activity and its relationship with alcohol consumption. In our study, the following three main results were reported: (a) only decreased PerAF differences in regional brain areas between patients with alcohol dependence and NCs were reported, including the left orbitofrontal cortex, right higher visual cortex, right supramarginal gyrus, right postcentral gyrus, and bilateral cerebellum posterior lobe. These brain areas may be interpreted as impaired regional functional activity caused by long-term alcoholism; (b) the regional brain areas that exhibited between-group differences exhibited extremely high discriminatory power in distinguishing the patients with alcohol dependence from the NCs. Therefore, the proposed PerAF may be served as a potential predictor to identify the alcohol-induced regional brain activity deficits; and (c) in the alcohol dependence group, the amount of daily alcohol consumption showed significant negative correlations with regional brain activity deficits.

Patients with alcohol dependence have been found to be associated with impaired balance and coordinating movement (31). Poor regulation of coordinating movement was one of the core characteristics of alcohol dependence (7, 8). The cerebellar circuits are associated with motor control and driving behavior, which have been shown to be impaired by alcohol dependence and alcohol intoxication (5, 7, 32, 33). The cerebellum posterior lobe is associated with the regulation of coordinating movement and is particularly vulnerable to alcoholism (13, 31, 34). Functional studies have shown altered regional neural brain activity (3, 8) and resting-state functional connectivity in the cerebellum in patients with alcohol dependence (5, 7, 35–37). Morphological studies also found decreased gray matter volumes in this area (38, 39), and this area may predict the relapse risk of alcoholism (40). Our results of decreased PerAF differences in the bilateral cerebellum posterior lobe were consistent with these findings. Furthermore, the extent of damage to the cerebellum positively correlated with the amount of daily alcohol consumption. These findings may be interpreted as functional impairment of the cerebellum caused by alcohol dependence.

The higher visual areas are divided into two distinct visual pathways, namely, the object and spatial property processing pathways (7, 29, 41–43). The spatial property processing pathway runs from the occipital lobe and up to the posterior parietal lobe, which is also essential for guiding movements. Damage to this pathway may disrupt the ability of visual location. The decreased functional connectivity in the higher visual cortex was reported in previous studies (5, 7). Chen et al. found decreased functional connectivity density of the visual pathway, and the visual pathway exhibited a high degree of discriminatory power with an extremely high sensitivity of 91.7% and specificity of 91.7% (7). In this study, decreased PerAF differences in the spatial property processing pathway were reported, which negatively correlated with the amount of daily alcohol consumption. In alcoholics, the concept of inefficiency includes difficulties in isolating irrelevant information (44), which is necessary for discriminating the targets from the distractors (5). Therefore, our findings of decreased PerAF differences in the spatial property processing pathway may reflect functional impairment of the visual pathway.

Alcoholism has been found to cause anatomical and functional damage to cognitive function, especially to verbal and spatial working memory (45, 46). Luo et al. and Tu et al. have reported impaired regional neural activity and functional connectivity of the orbitofrontal cortex, and the extent of damage of the orbitofrontal cortex positively correlated with years of drinking (5, 8). In this study, decreased PerAF difference in the orbitofrontal cortex was found in alcohol dependence compared with the NCs. The orbitofrontal cortex is thought to play an important role in the output of executive function and compulsive drug-seeking behaviors (47). In this framework, the decreased PerAF area in the orbitofrontal cortex may be an important etiology of executive function deficits and compulsive drug-seeking behaviors in patients with alcohol dependence.

## Limitations

However, some limitations should be noted. First, resting-state hemodynamic fluctuations (as well as EEG/MEG resting state activations) are prone to skills (e.g., musicians), personality, or psychiatric disorders. However, these factors are not considered in this study. Second, relatively small sample size was included. Third, some kind of psychiatric problems for patients with alcohol dependence is not mentioned in this study. Fourth, resting state “activations” are not activations in the true neurophysiological sense but hemodynamic fluctuations and may be associated with a couple of negative emotions.

## Conclusion

The proposed method of PerAF may be served as a potential sensitivity biomarker to identify alcohol-induced regional brain activity deficits. Our findings have shown altered regional brain activity deficits in the cerebellar-visual-orbitofrontal cortex with an extremely high degree of discriminatory power. These changes may be the etiology of executive function deficits, compulsive drug-seeking behaviors, and poor regulation of coordinating movement (e.g., driving behavior) in patients with alcohol dependence, which could expand our understanding of the pathophysiological mechanism of alcohol dependence.

## Data availability statement

The original contributions presented in the study are included in the article/Supplementary material, further inquiries can be directed to the corresponding author/s.

## Ethics statement

This study was approved by Ethical Committee of The Affiliated Huai'an No. 1 People's Hospital of Nanjing Medical University. The patients/participants provided their written informed consent to participate in this study. Written informed consent was obtained from the individual(s) for the publication of any potentially identifiable images or data included in this article.

## Author contributions

YW wrote the main manuscript text. YW and BS collected and analyzed the data, conceived and designed the whole experiment, and revised the manuscript. All authors contributed to the article and approved the submitted version.

## Conflict of interest

The authors declare that the research was conducted in the absence of any commercial or financial relationships that could be construed as a potential conflict of interest.

## Publisher's note

All claims expressed in this article are solely those of the authors and do not necessarily represent those of their affiliated organizations, or those of the publisher, the editors and the reviewers. Any product that may be evaluated in this article, or claim that may be made by its manufacturer, is not guaranteed or endorsed by the publisher.
