# Supplementary material for: Alcohol-induced brain deficit in alcohol dependence
Source: Front Neurol. 2022 Oct 25;13:1036164. doi: 10.3389/fneur.2022.1036164 (PMC9644208; doi:10.3389/fneur.2022.1036164)
Supplement: Supplementary file 1 [file Table_1.docx]

Supplementary Table 1 Characteristics of alcohol dependent and NCs

|  | Alcohol dependent | Healthy subjects | t/χ^2^ value | *p* value |
| --- | --- | --- | --- | --- |
| **Demographics** | | | | |
| Mean age, years | 48.62±6.81 | 48.48±7.05 | 0.076 | 0.94 |
| Sex (Male, Female) | 29 (20, 9) | 29 (18, 11) | 0.305^*^ | 0.581 |
| Education, years | 9.52±2.87 | 8.48±3.1 | 1.318 | 0.193 |
| Years of drink, years | 27.93±10.28 | N/A | N/A | N/A |
| SADQ score | 20.34±6.89 | N/A | N/A | N/A |
| AUDIT score | 23.83±5.55 | 2.55±0.95 | 20.35 | <0.001 |
| Daily alcohol consumption, ml | 239.66±107.22 | N/A | N/A | N/A |

**Note:** ^*^, χ^2^ test; Data are mean ± standard deviation values.

**Abbreviations:** NCs, Normal controls; SADQ, Severity of alcohol dependence questionnaire; AUDIT, Alcohol use disorders identification test; N/A, not applicable.
